# Global reach, equity, and learning outcomes of a digital surgical education platform: a RE-AIM implementation-effectiveness evaluation of SURGhub

**DOI:** 10.7189/jogh.16.04376

**Published:** 2026-10-30

**Authors:** Dáire Goodman, Musliu Adetola Tolani, Sara Mohan, Sebastian Hofbauer, Eric O’Flynn, Juan Carlos Puyana

**Affiliations:** 1 Institute of Global Surgery, School of Population Health, Royal College of Surgeons in Ireland, Dublin, Ireland; 2 Department of Surgery, Ahmadu Bello University Teaching Hospital, Zaria, Nigeria; 3 UCD Perinatal Research Centre, University College Dublin, National Maternity Hospital, Dublin, Ireland; 4 Global Surgery Foundation, Geneva, Switzerland; 5 Department of Surgery, University of Pittsburgh, Pittsburgh, Pennsylvania, USA

**Keywords:** global surgery, surgical education, e-learning, implementation science, RE-AIM, health equity

## Abstract

**Background:**

Healthcare providers in resource-constrained settings face persistent barriers to continuing professional development. Digital platforms may offer scalable approaches to surgical education, but evidence on implementation, equity, and learning outcomes at a global scale is limited. We evaluated the reach, implementation, equity, and learning outcomes of the United Nations Global Surgery Learning Hub (SURGhub) using the Reach, Effectiveness, Adoption, Implementation, and Maintenance framework.

**Methods:**

We conducted a cross-sectional evaluation of 112 SURGhub courses delivered between June 2023 and July 2025, alongside an uncontrolled paired analysis of learner knowledge acquisition. We analysed prospectively collected content provider, aggregated learner, and anonymised individual-level data to assess intervention outputs, reach, country-level uptake, implementation feasibility and acceptability, and changes in assessment scores.

**Results:**

SURGhub recorded 32,014 course enrolments from learners practising in 195 countries. The weighted mean progression and completion rates were 29.0% and 23.1%, respectively. Completion rates decreased from 34.0% among courses with shorter mean enrolment-to-completion intervals to 13.8% among those with longer intervals. Acceptability was high among course completers who responded to the survey, with a mean score of 4.57 on a five-point scale. A linear mixed-effects model including a random intercept for course estimated a mean increase in assessment score of 33.4 percentage points (95% confidence interval = 29.8–37.1) across 1,928 paired learner-course observations from 33 courses. Course-level mean increases ranged from 15.3 to 54.4 percentage points. Content providers were concentrated in high-income countries, while learners enrolled from both high-income and low- and middle-income settings, indicating broad geographic access along a geographic concentration of content production.

**Conclusions:**

SURGhub achieved global reach and high acceptability, demonstrating the potential of low-bandwidth, open-access digital platforms to deliver surgical education at scale. Completion should be interpreted alongside other engagement metrics, particularly for longer modular courses. Future research should assess long-term knowledge retention, clinical impact, multilingual content expansion, and participation by stakeholders from low- and middle-income countries.

Ten years after the Lancet Commission on Global Surgery, progress towards global surgery targets remains slow and uneven, particularly in resource-constrained settings (RCS). Unmet surgical need remains substantial: an estimated 160 million additional operations remain unperformed annually, while postoperative complications remain a major source of surgical morbidity worldwide [1–3]. Persistent gaps in workforce training and education requirements underscore the need for educational interventions tailored to healthcare providers in RCS [4]. Furthermore, significant disparities in access to high-quality, context-relevant digital surgical education persist in low- and middle-income settings (LMICs) despite expanding internet access [5].

The United Nations Global Surgery Learning Hub (SURGhub) is a joint initiative of the Global Surgery Foundation and United Nations Institute for Training and Research, supported by the Royal College of Surgeons in Ireland in association with the Johnson & Johnson Foundation. This community-led platform provides global surgery education tailored to the learning needs of healthcare providers in RCS [5,6]. This open-access platform and mobile application provide educational content in low-bandwidth environments and enable offline learning, thereby addressing key connectivity barriers to digital learning in RCS. An international multidisciplinary panel of experts in surgery, anaesthesia, obstetrics and gynaecology, and nursing reviews SURGhub content through a structured quality-assurance process that aims to maintain clinical accuracy and educational integrity [5]. As of June 2026, more than 50,000 learners from 195 countries had enrolled on SURGhub, through which they can access more than 140 courses intended for members of the surgical team [6].

The full scale and scope of SURGhub’s content or its implementation outcomes are yet to be characterised. Evidence on learning effectiveness, which is necessary for guiding the ongoing platform optimisation, also remains limited. We therefore characterised platform outputs and applied four of the five domains of the Reach, Effectiveness, Adoption, Implementation, and Maintenance (RE-AIM) framework to evaluate SURGhub’s implementation and learning outcomes [7]. To our knowledge, this is one of the largest evaluations of a global surgery digital learning platform and one of the first large-scale applications of the RE-AIM framework to online surgical education in a multinational cohort. This study integrates implementation metrics with learner-level outcomes and contributes evidence on scalable, low-bandwidth digital education for the global surgical workforce [7,8].

## METHODS

### Study design

We conducted a global evaluation of SURGhub using a cross-sectional design to assess equity of content production and implementation processes, alongside an uncontrolled, paired pre-course and post-course analysis of learner knowledge acquisition. The study is reported in accordance with the STROBE guideline for cross-sectional studies.

### The SURGhub intervention package

SURGhub is a community-led, open-access digital platform that provides multidisciplinary surgical education. All content undergoes structured review by the Project Scope, Technical, and Content Committees before publication to ensure its relevance, technical quality, and educational integrity [9].

### Target courses, population, and eligibility criteria

We included all 112 SURGhub courses delivered between June 2023 and July 2025 for which post-course data were available at the time of extraction in July 2025 in the descriptive analysis (Table S5 in the **Online Supplementary Document**). Of these, 33 courses also had pre-course and post-course knowledge assessment data and were included in the paired analysis of the Effectiveness domain, comprising 1928 learner-course observations. We excluded observations that lacked both a pre-course and post-course assessment for the same course. The de-identified data set did not contain a stable learner identifier, so individual learners could not be linked across courses.

### Study conceptual model

We selected the RE-AIM framework because it enables simultaneous evaluation of intervention effectiveness and implementation across diverse real-world health-system contexts [7,8]. We assessed the four domains as follows: Reach based on enrolment and geographic distribution; Effectiveness based on paired changes in knowledge scores; Adoption based on learner participation at the country level; and Implementation based on course progression, completion, time from enrolment to completion, and acceptability. We did not assess the Maintenance domain because the available data did not include longitudinal follow-up of sustained engagement, knowledge retention, or behaviour change.

### Data collection

We used prospectively collected content-provider data, aggregated learner data, and anonymised individual learner data routinely collected by SURGhub. The aggregated data set did not include information on learner sex or gender.

### Equity of content production

We defined equity of content production as the distribution of course content by the country of the content-providing organisation, representation of source institutions from high-income and low- and middle-income countries, and the speciality or subspeciality of the course content. We used these measures as proxies for equity in knowledge production on the SURGhub platform. The SURGhub Project Scope Committee categorised each course, and courses could be assigned to more than one speciality or subspeciality. These categories were therefore not mutually exclusive.

### Implementation process

Aggregated course-level data included enrolment, country of practice, progression beyond 50% of course content, completion and the mean time from enrolment to recorded completion. We interpreted progression and completion as indicators of structured engagement rather than comprehensive measures of learning, recognising that learners may derive educational value from partial engagement, particularly in longer or modular courses. The recorded interval did not distinguish between active learning, inactivity, pauses between sessions, and delayed return to a course.

We assessed the overall acceptability of the course implementation using aggregated responses on a five-point Likert scale across three post-course evaluation domains: overall course satisfaction, the relevance and engagement of course content, and the user-friendliness and reliability of the course platform.

### Learning outcomes

The Effectiveness domain data set included course-specific multiple-choice knowledge assessments. The same assessment was administered before and after all but one of the 33 courses, which used a different post-course assessment.

### Study outcomes and definitions

We assessed the Reach domain using course enrolments and the geographic distribution of unique learners, and the country-level Adoption domain based on learners’ self-reported country of practice. We assessed the Implementation feasibility domain using progression beyond 50% of the course content, course completion, and elapsed time from enrolment to completion, with completion defined using the platform-recorded course completion variable.

We categorised courses according to their mean elapsed time from enrolment to completion as short (≤60 minutes), medium (61–180 minutes), or long (>180 minutes). This measure included both active and inactive time, so we did not interpret it as active learning time.

We assessed the Implementation acceptability domain based on the mean rating across three post-course evaluation domains: overall satisfaction, content relevance and engagement, and platform usability and reliability. We defined the Effectiveness domain as the within-learner change in knowledge score, calculated as post-course minus pre-course score expressed in percentage points.

### Data management and missing data handling

We included 112 course records in the descriptive data set. The mean time from enrolment to completion was available for 111 courses. We excluded one course from the analysis by interval category. We restricted rankings of the highly rated courses to courses with at least 50 certificates issued to reduce instability associated with small denominators.

We included only learner-course observations with paired pre-course and post-course assessments in the Effectiveness analysis. We did not impute missing values. The de-identified data set did not contain a stable learner identifier that allowed learners to be linked across courses.

### Statistical analysis

We reported categorical variables using counts and percentages, and continuous variables using means and standard deviations or median and interquartile ranges, as appropriate. We calculated platform-level progression and completion as enrolment-weighted proportions, and implementation acceptability as mean ratings on a five-point Likert scale. We summarised enrolment, progression beyond 50%, and completion separately within the short, medium and long course groups. We assessed associations between mean elapsed time from enrolment to completion, course completion, and enrolment using Spearman’s rank correlation.

We summarised pre-course scores, post-course scores, and within-learner changes (Δ) by course, and assessed course-specific changes using paired *t*-tests. We estimated the overall mean change using a linear mixed-effects model with a random intercept for course to account for clustering within courses and conducted an intercept-only linear regression with course-clustered robust standard errors as a sensitivity analysis. We evaluated differences in improvement between courses using the Kruskal–Wallis test, followed by Dunn’s pairwise comparisons with Holm correction. We reported results with 95% confidence intervals (CIs). We performed all analyses in Stata, version 19.5 (StataCorp LLC, College Station, Texas, USA).

## RESULTS

### Implementation process outcomes

#### Reach

We recorded 32,014 course enrolments across the 112 SURGhub courses. The five courses with the highest enrolment, which accounted for 10,467 (32.7%) of all enrolments, were Surgical Foundations (n = 3,422), Research Methodology (n = 2262), Essential Emergency and Critical Care (n = 2,049), Trauma and Disaster Team Response (n = 1,706), and the PeN Programme (n = 1,028). Three of these five courses – Surgical Foundations, Research Methodology, and the PeN Programme – were regional or partnership-based programme-style courses (Table S3 in the **Online Supplementary Document**).

#### Country-level learner adoption

The 32,014 course enrolments represented 11,054 unique learners from 195 countries (**Figure 1**). The five countries with the largest learner populations were India (n = 1,106), the USA (n = 820), the UK (n = 658), Kenya (n = 538), and Nigeria (n = 451).

**Figure 1:**
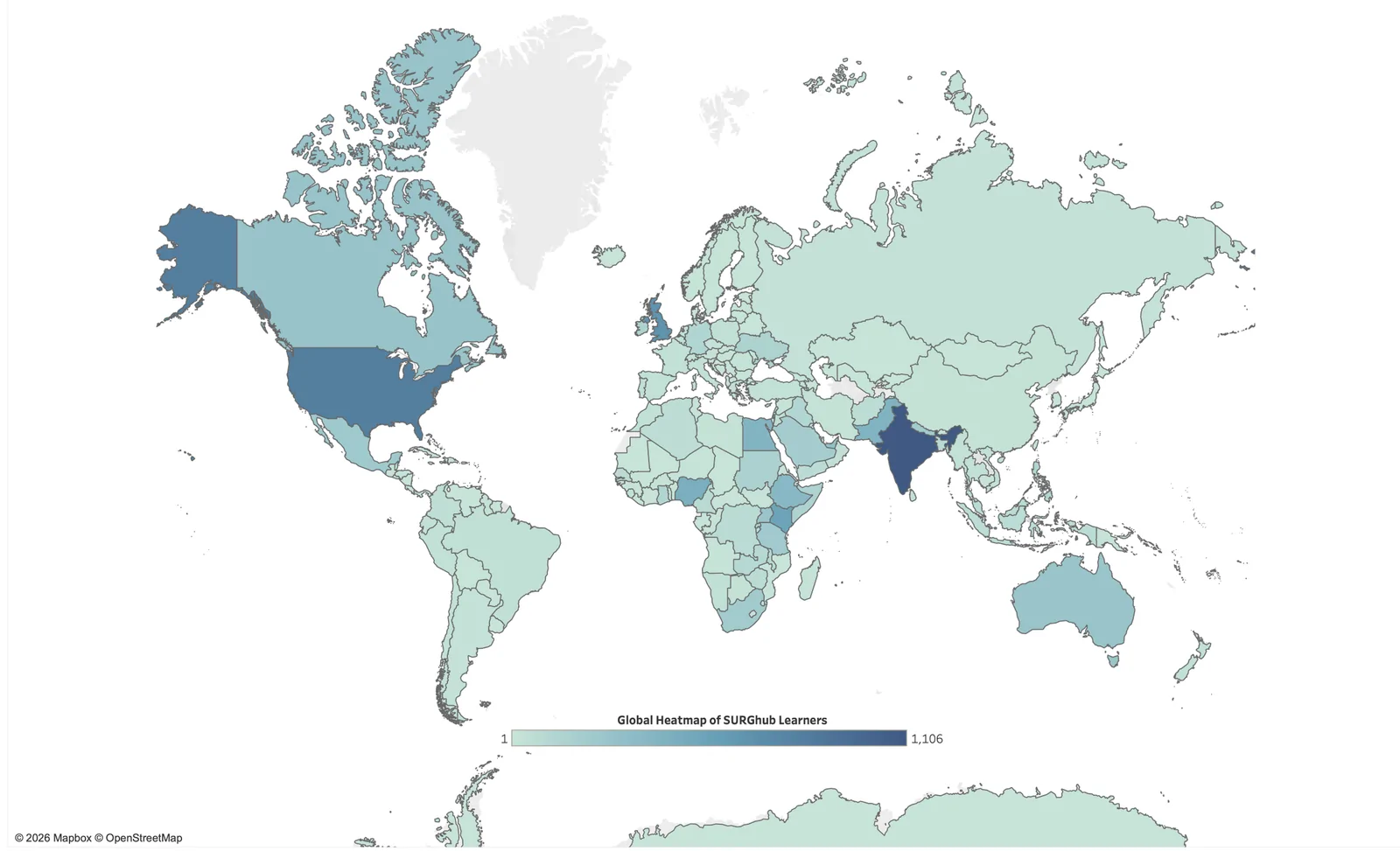
Geographic distribution of unique SURGhub learners by self-reported country of practice. Countries are shaded according to the number of learners enrolled during the study period. SURGhub – United Nations Global Surgery Learning Hub.

#### Implementation feasibility

The enrolment-weighted proportion of learners who progressed beyond 50% of the course content was 29.0%, and the enrolment-weighted completion rate was 23.1%, with variation in completion rates across courses (Table S4 in the **Online Supplementary Document**). The mean time from enrolment to completion was available for 111 of 112 courses. We categorised 56 courses as short (≤60 minutes), 37 as medium (61–180 minutes), and 18 as long (>180 minutes). The completion rate decreased from 34.0% among short courses to 25.4% among medium courses and 13.8% among long courses. The corresponding proportions of learners who progressed beyond 50% were 42.7%, 32.5% and 16.8%, respectively. Mean elapsed time was negatively correlated with completion (Spearman’s ρ = −0.514, *P* < 0.001) and positively correlated with enrolment (Spearman’s ρ = 0.355, *P* < 0.0001) (**Table 1**).

**Table 1:** Engagement metrics by mean elapsed enrolment-to-completion interval

Course category based on mean elapsed interval	Number of courses	Number of enrolments	Weighted progression, %	Weighted completion, %
Short (≤60 min)	56	9,068	42.7	34.0
Medium (61–180 min)	37	9,932	32.5	25.4
Long (>180 min)	18	12,989	16.8	13.8

#### Implementation acceptability

Among responding course completers, the mean overall implementation acceptability score was 4.57 out of 5. Ratings were consistently high across all three domains, with overall course satisfaction being 4.59, content relevance and engagement being 4.54, and platform usability being 4.59. Among courses that issued at least 50 certificates, the highest-rated courses included Essential Burn Care, Introduction to C-Arm Imaging and Radiation Safety in the Operating Room, Infection Control and Prevention 2023, Hand Trauma Reconstruction, and Microsurgery and Workhorse Flaps (**Table 2**).

**Table 2:** Implementation acceptability scores for selected highly rated courses and for SURGhub overall

Course	Number of times course was completed	Overall course satisfaction, x̄	Content relevance and engagement, x̄	Platform usability and reliability, x̄	Overall implementation acceptability, x̄
Essential Burn Care	275	4.70	4.70	4.70	4.70
Introduction to C-Arm Imaging and Radiation Safety in the Operating Room	179	4.70	4.70	4.70	4.70
Infection Control and Prevention 2023	135	4.60	4.70	4.80	4.70
Hand Trauma Reconstruction	114	4.70	4.70	4.70	4.70
Microsurgery and Workhorse Flaps	93	4.70	4.70	4.70	4.70
SURGhub overall	NA	4.59	4.54	4.59	4.57

NA – not applicable, SURGhub – United Nations Global Surgery Learning Hub, x̄ – mean

#### Equity of content production

Across 112 courses, anaesthesia was the most frequently assigned speciality (n = 52), followed by plastic and reconstructive surgery (n = 27) and nursing (n = 23). Orthopaedic surgery, general surgery, and trauma and critical care were each assigned to 18 courses, while obstetrics and gynaecology was assigned to 17 courses (Table S1 in the **Online Supplementary Document**).

Courses were most frequently submitted by organisations based in the USA (n = 46) and UK (n = 43), followed by Ethiopia (n = 9), Mexico (n = 8), Ireland (n = 6), and Australia (n = 6) (**Figure 2**). The World Federation of Societies of Anaesthesiologists contributed 29 courses (25.9%), while ReSurge International contributed 20 (17.9%) (Table S2 in the **Online Supplementary Document**).

**Figure 2:**
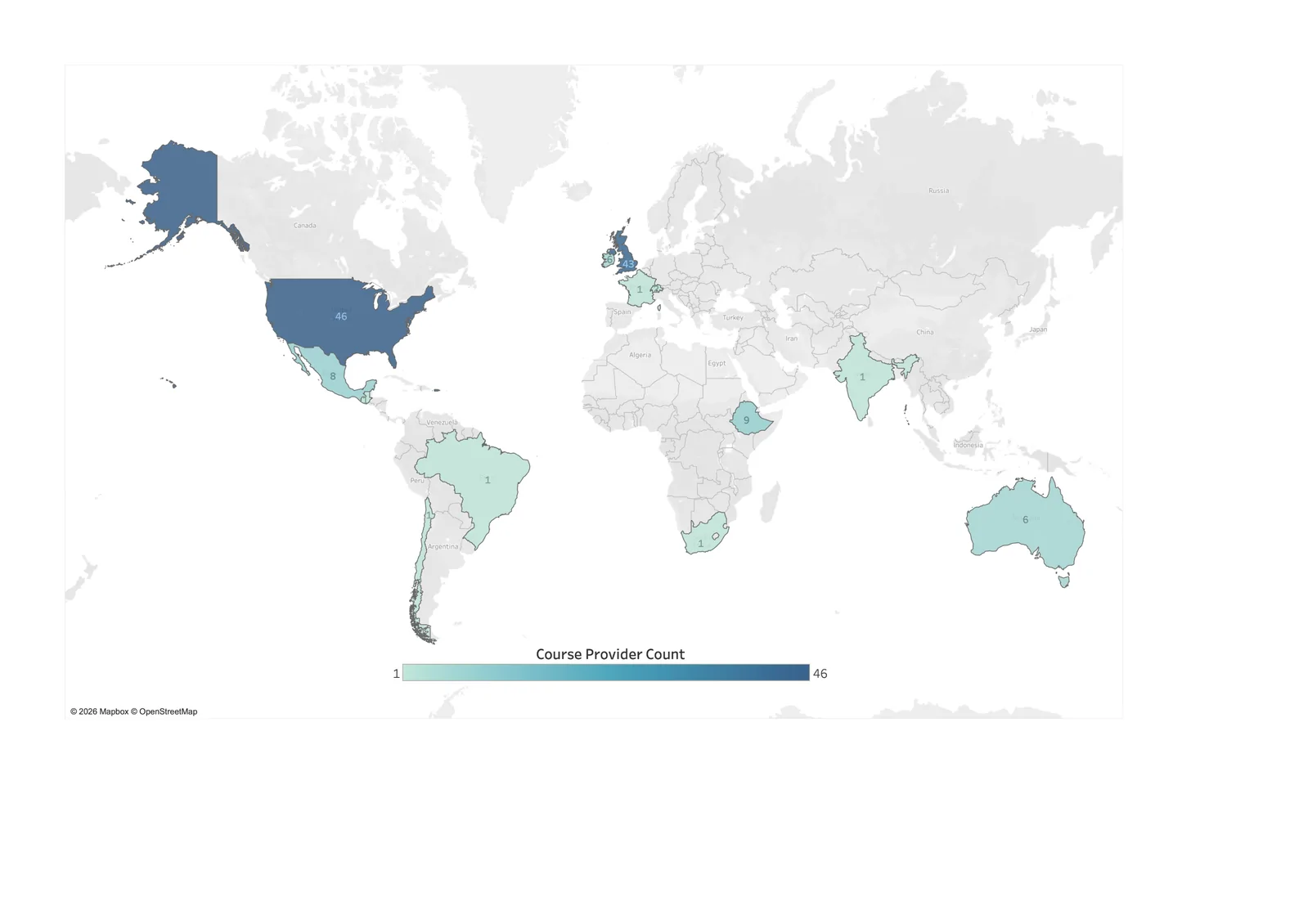
Geographic distribution of organisations providing courses through SURGhub. Countries are shaded according to the number of courses contributed by organisations based in each country during the study period. Darker shading indicates a greater number of contributed courses, while countries without recorded contributions are shown in grey. SURGhub – United Nations Global Surgery Learning Hub.

### Learning outcomes

#### Effectiveness

Using a random intercept model, we estimated an overall mean improvement in knowledge scores of 33.4 percentage points (95% CI = 29.8–37.1, *P* < 0.001) across 1928 paired learner-course observations from 33 courses. The course-level intraclass correlation coefficient was 0.088 (95% CI = 0.048–0.153), indicating that 8.8% of the variance in score improvement was attributable to differences between courses. A sensitivity analysis using course-clustered robust standard errors produced a similar estimate of 33.1 percentage points (95% CI = 29.1–37.2, *P* < 0.001).

We observed course-level mean improvements ranging from 15.3 to 54.4 percentage points (**Figure 3**; Table S6 in the **Online Supplementary Document**). We found increases in mean scores across all courses, although the 95% confidence intervals included zero for Chest Wall Reconstruction and Ectopic Pregnancy, both of which had small paired samples.

**Figure 3:**
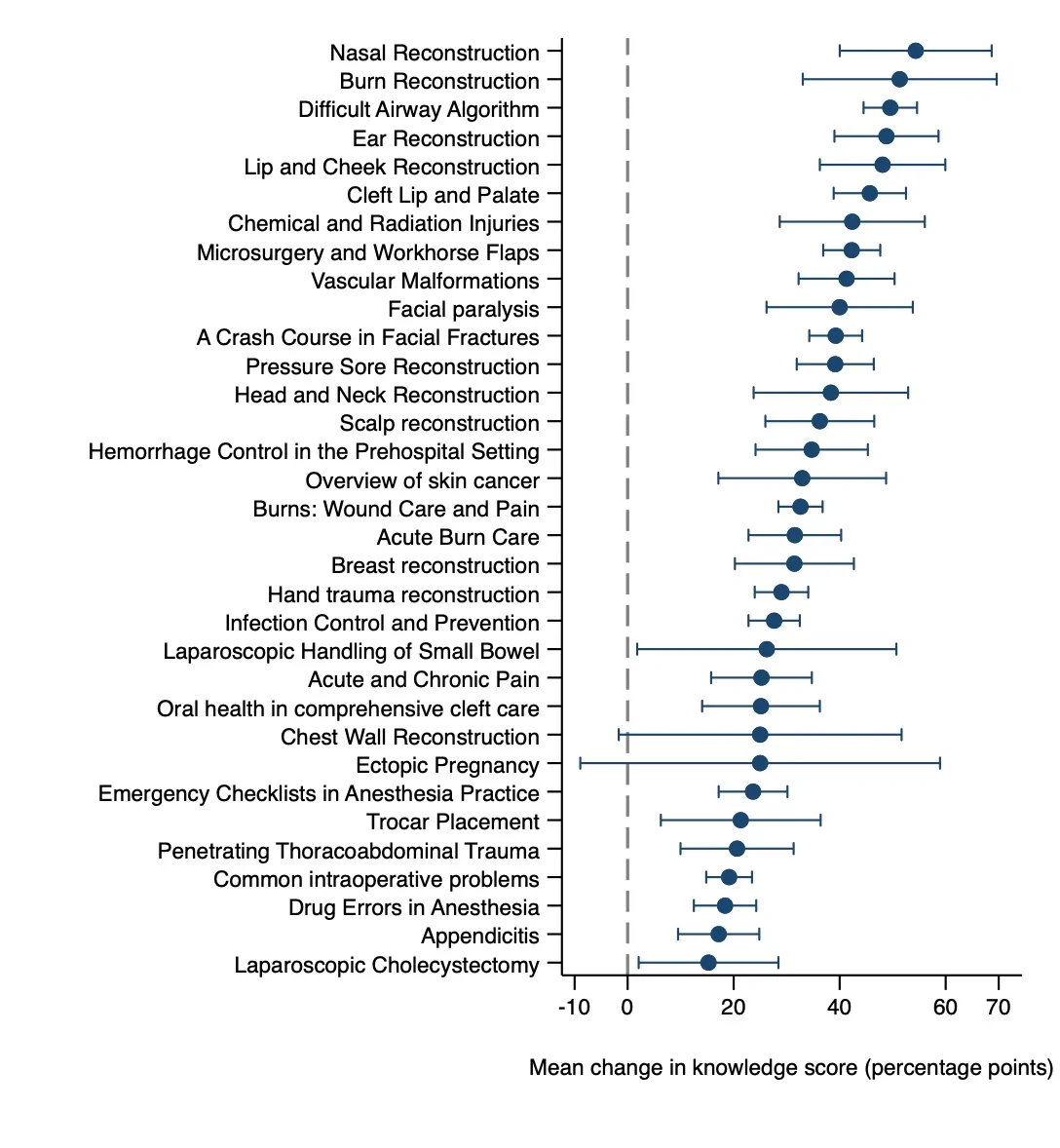
Immediate within-learner changes in knowledge scores across 33 SURGhub courses. Points represent course-specific mean percentage-point changes between pre-course and post-course knowledge assessments, while horizontal lines represent 95% confidence intervals. Courses are ordered by mean change. Assessments were course-specific and were not standardised across courses; therefore, between-course differences should not be interpreted as rankings of course effectiveness. CI – confidence interval, SURGhub – United Nations Global Surgery Learning Hub.

We found significant changes in knowledge scores among courses (Kruskal-Wallis *H* = 205.8, df = 32, *P* < 0.001), with a small-to-moderate effect size (η^2^*_H_*   = 0.092). Holm-adjusted Dunn *post hoc* comparisons identified multiple between-course differences, providing further evidence of heterogeneity in learning outcomes.

## DISCUSSION

This evaluation identified three main findings. First, SURGhub achieved broad reach, achieving 32,014 course enrolments representing 11,054 unique learners from 195 countries. Second, implementation acceptability was high among course completers who responded, while learners with paired assessments showed substantial immediate improvements in knowledge scores. Third, broad learner adoption was not matched by comparable equity in content production, which remained concentrated among organisations based in high-income countries. Together, these findings provide evidence across the Reach, Effectiveness, Adoption and Implementation domains of the RE-AIM framework [7,8].

Our findings suggest that low-bandwidth digital education can help strengthen the global surgical workforce, which is particularly relevant due to the slow progress towards global surgery targets and the increasing recognition of surgical services as essential health-system infrastructure [3]. However, broad learner access was not accompanied by comparable equity in content production. Courses were disproportionately produced by organisations based in high-income countries, particularly the USA and the UK, with comparatively limited contribution from French-, Portuguese- and Spanish-speaking countries. This pattern suggests both linguistic and geographical imbalances in content development, consistent with the continued dominance of English-language resources in open-access medical education [10].

### Equity of content provision

The predominance of content providers from high-income countries reflects broader structural inequities in global health knowledge production and dissemination, as authorship, funding, and agenda-setting remain concentrated in high-income settings [11–13]. Although SURGhub achieved broad learner adoption, content provision remained concentrated among organisations based in high-income countries, particularly the USA and the UK, highlighting the distinction between equity of access and equity of content provision. However, the country in which the provider organisation was based served only as a proxy for equity in knowledge production and did not capture individual authorship, leadership, or co-creation processes. Furthermore, these categories were not mutually exclusive, as courses could be assigned to more than one speciality.

Addressing these imbalances will require intentional strategies, including co-creation with LMIC partners, expansion of regional content hubs, language localisation, and development of context-specific educational materials. Surgical Foundations, Research Methodology, and the PeN Programme were among the courses with the highest enrolment, although regional and partnership-based programmes represented a minority of course offerings. This finding suggests demand for regionally relevant, modular content and supports further investment in content developed with or led by partners in LMICs and regional training organisations.

### Implementation and engagement

SURGhub achieved substantial reach, with more than 32,000 enrolments across 112 courses and a broad geographic distribution of learners. Previous studies have reported greater use of free open-access medical education resources in high-income countries [14,15]. In comparison, SURGhub attracted considerable participation from learners in both LMICs and high-income countries. The overall course completion rate was 23.1%, which is consistent with rates reported for massive open online courses. These courses commonly achieve completion rates ranging from less than 10% to approximately 30%, depending on course design, learner motivation, and engagement [16,17].

Courses with longer mean intervals between enrolment and completion had lower completion rates. However, this measure included both active and inactive time and therefore does not show that longer active study reduced the likelihood of completion. This limitation particularly affects the interpretation of longitudinal programmes such as Surgical Foundations, which is designed to be completed over two years [18]. Completion represents only one dimension of engagement, especially for modular or programme-based courses that learners may access over an extended period.

Barriers such as limited time, clinical workload, and inconsistent internet access likely affect engagement in digital education [10,19,20]. Nevertheless, respondents rated the implementation acceptability of the SURGhub courses highly, consistent with previous reports of strong learner satisfaction with online surgical education platforms [21].

### Learning outcomes

Using a random intercept model, we estimated an overall immediate improvement in knowledge scores of 33.4 percentage points within the RE-AIM Effectiveness domain. We arrived at a similar estimate in a sensitivity analysis using course-clustered robust standard errors. We also observed variation in improvement across courses, which could be explained by differences in baseline knowledge, course design, assessment difficulty, content specificity, or alignment with learners’ clinical practice. Nevertheless, these results suggest that SURGhub can support short-term knowledge acquisition at scale. Future studies should assess knowledge retention, translation into clinical practice, and contributions to workforce capacity building.

### Implications for global surgical education

These findings have implications for global surgical workforce development. SURGhub demonstrates the potential of low-bandwidth digital platforms to expand access to education beyond traditional training infrastructures, aligning with the workforce priorities outlined by the Lancet Commission on Global Surgery [1].

This study also supports a portfolio approach to scalable digital education. High-enrolment foundational courses may provide entry points for learners; shorter, clinically focused ones could have higher completion rates, while longer programme-style courses can provide structured curricula that learners access over time. A combination of foundational, practical, and contextually relevant programme-style content may therefore meet different learner needs more effectively than a uniform approach to course design.

### Strengths and limitations

This study has several strengths, including its large multinational data set spanning 195 countries and an evaluation of real-world implementation rather than a pilot intervention. The platform’s multidisciplinary scope, inclusion of implementation and learning outcomes, and the application of the RE-AIM framework strengthen the practical relevance and transferability of the findings.

However, several limitations should be considered. The single-group pre–post design lacked a control group, which limited causal inference, as regression to the mean and broader contextual influences may partly explain the observed improvements [22,23]. Most courses used the same pre-course and post-course assessment, which may have introduced test–retest familiarity or test-enhanced learning effects [24]. Assessments were taken immediately after course completion, so the results provide no evidence of longer-term knowledge retention [25]. Assessments were course-specific rather than standardised assessments, which limited direct interpretation of between-course differences in score improvement. The geographic data set identified 11,054 unique learners, but we could not link these learners to the individual course data sets and therefore could not determine the distribution of courses taken per learner. We also could not link learners’ countries of practice or modes of platform access to individual course outcomes, so we could not compare implementation and learning outcomes by country income group or offline application use.

We analysed effectiveness only among learners who completed paired assessments, potentially introducing selection bias towards more engaged or motivated learners and limiting generalisability of the findings to non-completers. The de-identified Effectiveness domain data set did not contain a stable learner identifier across courses. The analysis accounted for clustering within courses, but we could not identify learners who participated in multiple courses or account for possible correlations between observations from the same learner across different courses.

The data set provided several implementation metrics, including elapsed enrolment-to-completion interval and Likert ratings, only as aggregate course-level data. We assessed implementation acceptability only among course completers who submitted a post-course evaluation, so the findings may not reflect the views of all enrolled learners. The elapsed enrolment-to-completion interval included both active and inactive time, and prolonged inactive log-in periods or offline access to download materials may have affected this measure. Finally, we did not assess the RE-AIM Maintenance domain because the available data captured immediate implementation and post-course learning outcomes rather than longer-term retention, behaviour change, or sustained engagement [26]. Despite these limitations, the broad geographic reach, high acceptability, and observed knowledge gains support SURGhub as a scalable model for global surgical education.

### Future directions

These findings provide an empirical basis for future content commissioning, translation strategies, and partner engagement. High-enrolment foundational courses may serve as anchors for attracting new learners and expanding curriculum coverage, and the higher completion rates in procedural and safety-oriented courses support concise, clinically relevant course design. Expanding non-English content and increasing participation from organisations in LMICs may further improve adoption, contextual relevance, and equity in knowledge production.

## CONCLUSIONS

SURGhub represents a scalable, open-access digital education platform that can deliver surgical education globally, with substantial reach, broad adoption across countries, high learner acceptability, and consistent improvements in knowledge among a diverse international cohort. Our findings point to the potential of a low-bandwidth digital educational model to support global surgical workforce development, particularly in resource-constrained settings. They also highlight the disparity between equity of access and equity of content provision. Expanding multilingual content and increasing leadership among organisations based in LMICs will be essential for improving contextual relevance and representation in global surgical education. Future research should evaluate long-term outcomes, including knowledge retention, behavioural change, and impact on clinical practice, and identify determinants of learner engagement and completion to further optimise implementation of digital education platforms in real-world settings.

## Additional material


Online Supplementary Document

## Data Availability

**Data availability:** The de-identified data underlying the findings of this study are available from the corresponding author upon reasonable request, subject to approval by the relevant data controller and compliance with applicable ethical, data-protection, and institutional requirements. The data are not publicly available because they comprise individual-level educational records governed by institutional data-sharing restrictions.
