# Supplementary material for: Global reach, equity, and learning outcomes of a digital surgical education platform: a RE-AIM implementation-effectiveness evaluation of SURGhub
Source: J Glob Health. 2026 Oct 30;16:04376. doi: 10.7189/jogh.16.04376 (PMC13721840; doi:10.7189/jogh.16.04376)
Supplement: Supplementary file 1 [file jogh-16-04376-s001.pdf]

Supplement to: Goodman D, Tolani MA, Mohan S, Hofbauer S, O'Flynn E, Puyana JC. Global reach, equity, and learning outcomes of a digital surgical education platform: a RE-AIM implementation-effectiveness evaluation of SURGhub. J Glob Health. 2026;16:04376.

Table S1: Distribution of courses hosted on the SURGhub platform by speciality

**Speciality**	**Frequency of courses***
Anaesthesia	52
Plastic and reconstructive surgery	27
Nursing	23
Orthopaedic surgery	18
General surgery	18
Trauma and critical care surgery	18
Obstetrics & Gynaecology	17
Emergency Medicine	11
Oral and Maxillofacial (dental) surgery	8
Otorhinolaryngology (ENT)	7
Vascular surgery	5
Paediatric surgery	3
Cardiothoracic surgery	2
Urology	2
Neurosurgery	1

*Speciality categories were not mutually exclusive. Courses could be assigned to more than one speciality or subspeciality; therefore, counts do not sum to 112.

Table S2: Top five submitting organisations of the courses hosted on the SURGhub learning platform

**Course Provider**	**Frequency of courses, n (%)***
World Federation of Societies of Anaesthesiologists (WFSA)	29 (25.9%)
ReSurge International	20 (17.9%)

TecSalud	8 (7.1%)
All-Safe	6 (5.4%)
Ausmed	5 (4.5%)
Behind the Knife	5 (4.5%)

*A denominator of 112 courses was used.

Table S3: Top five courses with the highest learner enrolment on the SURGhub platform

Course Name with Provider	Enrolments, n	Time to course completion (minutes)*
Surgical Foundations (COSECSA)	3422	3935
Research Methodology Course (CANECSA)	2262	134
Essential Emergency & Critical Care (EECC)	2049	314
Trauma and Disaster Team Response (McGill Centre for Global Surgery)	1706	229
PeN Programme (RCSI & ECSACONM)	1028	1317

*Only data on the aggregated mean time to course completion was available for each course

COSECSA = College of Surgeons of East, Central and Southern Africa, CANECSA = College of Anaesthesiologists of East, Central and Southern Africa, EECC = Essential Emergency and Critical Care, RCSI = Royal College of Surgeons in Ireland, ECSACONM = East, Central, and Southern Africa College of Nursing and Midwifery, PeN = Perioperative Nursing E-learning Foundational Programme

Supplemental Table 4: Top five courses with the highest completion rates on the SURGhub platform

Course Name and Provider	Positive Course Progression rate, n (%)	Completion rate, n (%)	Mean Time to course completion (minutes)
Personal Protective Equipment (Lifebox)	497 (70.9%)	408 (58.2%)	9
Rapid Sequence Induction (WFSA)	149 (63.4%)	127 (54.0%)	37
Pulse Oximetry (Lifebox)	132 (51.2%)	131 (50.8%)	59
Communication Skills for Telephone Consultation (RCSI)	89 (53.3%)	81 (48.5%)	65
Chest Wall Reconstruction (ReSurge)	9 (52.9%)	8 (47.1%)	49

WFSA = World Federation of Societies of Anaesthesiologists, RCSI = Royal College of Surgeons in Ireland

Supplemental Table 5: Courses hosted on the SURGhub Platform

Course Author	Country of Submitting Organisation	Course Name
All-Safe	United States, Ethiopia	Laparoscopic handling of Small Bowel
All-Safe	United States, Ethiopia	Trocar Placement
All-Safe	United States, Ethiopia	Penetrating Thoracoabdominal Trauma

All-Safe	United States, Ethiopia	Ectopic Pregnancy
All-Safe	United States, Ethiopia	Laparoscopic Cholecystectomy
All-Safe	United States, Ethiopia	Appendicitis
Amosmile	South Africa	Z-plasty
AO Alliance	United States	Introduction to C-Arm Imaging and Radiation Safety in the Operating Room
ASA	United States	Difficult Airway Algorithm 2023
ASA	United States	Infection Control and Prevention 2023
ASA	United States	Emergency Checklists in Anaesthesia Practice
ASA	United States	Drug Errors in Anaesthesia 2023
Ausmed	Australia	CVAD Complications: Prevention and Management
Ausmed	Australia	Preoperative Assessment
Ausmed	Australia	Postoperative Assessment and Monitoring
Ausmed	Australia	All About Aseptic Technique
Ausmed	Australia	Central Venous Access Devices (CVADs)
Baylor	United States	Chemical and Radiation Injuries
Behind the Knife	United States	Femoral Vessels
Behind the Knife	United States	Suturing - Horizontal Mattress
Behind the Knife	United States	Suturing - Vertical Mattress
Behind the Knife	United States	2-Handed Knot-Tying

Behind the Knife	United States	1-Handed Knot-Tying
CANECSA	Tanzania	Research Methodology Course
COSECSA	Tanzania	Surgical Foundations
CrashSavers	Guatemala, Chile, United States of America, Brazil	Haemorrhage Control in the Prehospital Setting
Ecancer	United Kingdom	Pancreatic Cancer Surgery Course
Ecancer	United Kingdom	Breast Cancer Surgery Course
Ecancer	United Kingdom	Colon Cancer Surgery Course
Ecancer	United Kingdom	Rectal Cancer Surgery Course
EECC	United States	Essential Emergency & Critical Care
F2AR	France	Apprendre et Transmettre l'Anesthésie Locorégionale Echoguidée en toute Sécurité
GSF	Switzerland	Exploring Perioperative Nursing
GSF	Switzerland	Navigating the Global Surgery Ecosystem
Interburns	United Kingdom	Essential Burn Care
Interburns	United Kingdom	Soins Fondamentaux en Brûlologie
International Federation of Rural Surgeons	India	Rural Urology Practice
King's College WHO Checklist	United Kingdom	Liste de Contrôle de l'OMS pour une Chirurgie Sûre
Lifebox	United Kingdom, United States, Ethiopia	Pulse Oximetry

Lifebox	United Kingdom, United States, Ethiopia	Pulse Oximetry in the context of COVID-19
Lifebox	United Kingdom, United States, Ethiopia	Personal Protective Equipment
McGill Centre for Global Surgery	United States	Trauma and Disaster Team Response
McGill Centre for Global Surgery	United States	Intervention de Traumatologie et de Catastrophe en Equipe
PerioperativeCPD	United Kingdom	Understanding Capnography
PerioperativeCPD	United Kingdom	Understanding Local Anaesthetic Systemic Toxicity (LAST)
PerioperativeCPD	United Kingdom	Understanding Arterial Blood Pressure Measurement
RCSI	Ireland	Video Consultations
RCSI	Ireland	Communication Skills for Telephone Consultation
RCSI	Ireland	Safe Clinical Handover
RCSI	Ireland	Patient-Centred Care
RCSI & ECSACONM	Ireland, Tanzania	PeN Programme (Spanish)
RCSI & ECSACONM	Ireland, Tanzania	PeN Programme
ReSurge	United States	Burn Reconstruction
ReSurge	United States	Nasal Reconstruction
ReSurge	United States	Ear Reconstruction
ReSurge	United States	Lip & Cheek Reconstruction
ReSurge	United States	Cleft Lip and Palate

ReSurge	United States	Microsurgery and Workhorse Flaps
ReSurge	United States	Vascular Malformations
ReSurge	United States	Facial Paralysis
ReSurge	United States	A Crash Course in Facial Fractures
ReSurge	United States	Overview of Skin Cancer
ReSurge	United States	Scalp Reconstruction
ReSurge	United States	Head and Neck Reconstruction
ReSurge	United States	Acute Burn Care
ReSurge	United States	Pressure Sore Reconstruction
ReSurge	United States	Breast Reconstruction
ReSurge	United States	Burns: Wound Care and Pain Management
ReSurge	United States	Hand Trauma Reconstruction
ReSurge	United States	Chest Wall Reconstruction
ReSurge	United States	Acute and Chronic Pain
ReSurge	United States	Common Intraoperative Problems
Safe Surgery Innovation	United Kingdom, United States	Surgical Instrument Testing and Inspection
Smile Train	United States	Oral Health in Comprehensive Cleft Care
TecSalud	Mexico	Lavado de Manos, Asepsia, Desinfección y Esterilización
TecSalud	Mexico	Manejo Básico de Quemaduras
TecSalud	Mexico	Técnica de Suturas y Puntos
TecSalud	Mexico	Manejo Básico de Heridas
TecSalud	Mexico	Suture Technique

TecSalud	Mexico	Hand Washing, Asepsis, Disinfection and Sterilization
TecSalud	Mexico	Basic Burn Management
TecSalud	Mexico	Basic Principles of Wound Management
University of Western Australia	Australia	Introduction to Musculoskeletal Tumour Surgery
WFSA	United Kingdom	Conversión de Analgesia Epidural para Labor a Anestesia para Operación Cesárea
WFSA	United Kingdom	Anestesia para Craneotomía y Resección de Tumor Cerebral
WFSA	United Kingdom	Transversus Abdominis Plane Block
WFSA	United Kingdom	Nerve Stimulation for Peripheral Nerve Blockade
WFSA	United Kingdom	Anaesthesia for Posterior Cranial Fossa Surgery
WFSA	United Kingdom	The Labour Epidural-Troubleshooting
WFSA	United Kingdom	Consideraciones Anestésicas para el Parto Vaginal luego de Cesárea
WFSA	United Kingdom	Anaesthetic Considerations for Vaginal Birth After Caesarean Delivery
WFSA	United Kingdom	Conversion of Labour Epidural Analgesia to Anaesthesia for Caesarean Delivery
WFSA	United Kingdom	Rapid Sequence Induction
WFSA	United Kingdom	Blood Sparing Techniques in Paediatric Anaesthesia

WFSA	United Kingdom	Tranexamic Acid
WFSA	United Kingdom	Acide Tranexamique
WFSA	United Kingdom	Anaesthesia for Caesarean Delivery
WFSA	United Kingdom	Lumbar Plexus Block: Landmark Technique (PSOAS Compartment Block)
WFSA	United Kingdom	Adjuvant Medications for Peripheral Nerve Blocks
WFSA	United Kingdom	Epidural en el Trabajo de Parto - Solución de Problemas
WFSA	United Kingdom	Fascia Iliaca Compartment Block: Landmark and Ultrasound Approach
WFSA	United Kingdom	Obstetrics in the Setting of Remote Retrieval Medicine
WFSA	United Kingdom	Perioperative Blood Management in the Pediatric Patient
WFSA	United Kingdom	Fascia Iliaca Compartment Block: An Update
WFSA	United Kingdom	Key concepts in perioperative management of spinal cord injuries
WFSA	United Kingdom	Perioperative Medicine: An Overview
WFSA	United Kingdom	Anaesthesia for Tissue Free Flap
WFSA	United Kingdom	Principles of Regional Anaesthesia in Children
WFSA	United Kingdom	Ultrasound Guided Femoral Nerve Block
WFSA	United Kingdom	Speaking up in the Operating Room - Escalating Concerns

WFSA	United Kingdom	Practical Considerations to Access the Epidural Space
WFSA	United Kingdom	Anaesthesia for Craniotomy and Brain Tumour Resection

Appendix Table 6: Full paired analysis of pre- and post-course knowledge scores (all courses)

**Course**	**n**	**Mean Δ**	**95% CI**	**p-value**
Nasal Reconstruction	23	54.4	40.0 to 68.7	<0.001
Burn Reconstruction	12	51.3	33.1 to 69.6	<0.001
Difficult Airway Algorithm	95	49.6	44.5 to 54.6	<0.001
Ear Reconstruction	52	48.8	39.0 to 58.7	<0.001
Lip and Cheek Reconstruction	21	48.1	36.3 to 59.9	<0.001
Cleft Lip and Palate	64	45.7	38.9 to 52.5	<0.001
Chemical and Radiation Injuries	24	42.4	28.7 to 56.1	<0.001
Microsurgery and Workhorse Flaps	101	42.3	36.9 to 47.7	<0.001
Vascular Malformations	59	41.3	32.3 to 50.4	<0.001
Facial Paralysis	36	40	26.2 to 53.8	<0.001
A Crash Course in Facial Fractures	173	39.3	34.3 to 44.2	<0.001
Pressure Sore Reconstruction	60	39.2	31.9 to 46.4	<0.001
Head and Neck Reconstruction	23	38.4	23.8 to 52.9	<0.001
Scalp Reconstruction	56	36.3	26.0 to 46.5	<0.001

Haemorrhage Control in the Prehospital Setting	36	34.7	24.1 to 45.3	<0.001
Overview of Skin Cancer	17	32.9	17.1 to 48.8	<0.001
Burns: Wound Care and Pain	180	32.6	28.4 to 36.8	<0.001
Acute Burn Care	52	31.5	22.8 to 40.3	<0.001
Breast Reconstruction	48	31.5	20.2 to 42.7	<0.001
Hand Trauma Reconstruction	133	29	24.0 to 34.1	<0.001
Infection Control and Prevention	160	27.6	22.8 to 32.5	<0.001
Laparoscopic Handling of Small Bowel	8	26.3	1.8 to 50.7	0.039
Acute and Chronic Pain	40	25.3	15.8 to 34.8	<0.001
Oral Health in Comprehensive Cleft Care	32	25.2	14.1 to 36.3	<0.001
Chest Wall Reconstruction	9	25	−1.7 to 51.7	0.063
Ectopic Pregnancy	10	25	−8.9 to 58.9	0.13
Emergency Checklists in Anaesthesia Practice	89	23.7	17.2 to 30.2	<0.001
Trocar Placement	15	21.3	6.3 to 36.4	0.009
Penetrating Thoracoabdominal Trauma	31	20.7	10.0 to 31.3	<0.001
Common Intraoperative Problems	140	19.1	14.8 to 23.5	<0.001
Drug Errors in Anaesthesia	64	18.4	12.5 to 24.3	<0.001
Appendicitis	46	17.2	9.5 to 24.8	<0.001
Laparoscopic Cholecystectomy	19	15.3	2.1 to 28.5	0.026

Supplemental Table 6: Full paired analysis of pre- and post-course knowledge scores (all courses)

Course	n	Mean Δ	95% CI	p-value
Nasal Reconstruction	23	54.4	40.0 to 68.7	<0.001
Burn Reconstruction	12	51.3	33.1 to 69.6	<0.001
Difficult Airway Algorithm	95	49.6	44.5 to 54.6	<0.001
Ear Reconstruction	52	48.8	39.0 to 58.7	<0.001
Lip and Cheek Reconstruction	21	48.1	36.3 to 59.9	<0.001
Cleft Lip and Palate	64	45.7	38.9 to 52.5	<0.001
Chemical and Radiation Injuries	24	42.4	28.7 to 56.1	<0.001
Microsurgery and Workhorse Flaps	101	42.3	36.9 to 47.7	<0.001
Vascular Malformations	59	41.3	32.3 to 50.4	<0.001
Facial Paralysis	36	40	26.2 to 53.8	<0.001
A Crash Course in Facial Fractures	173	39.3	34.3 to 44.2	<0.001
Pressure Sore Reconstruction	60	39.2	31.9 to 46.4	<0.001
Head and Neck Reconstruction	23	38.4	23.8 to 52.9	<0.001
Scalp Reconstruction	56	36.3	26.0 to 46.5	<0.001
Haemorrhage Control in the Prehospital Setting	36	34.7	24.1 to 45.3	<0.001
Overview of Skin Cancer	17	32.9	17.1 to 48.8	<0.001
Burns: Wound Care and Pain	180	32.6	28.4 to 36.8	<0.001
Acute Burn Care	52	31.5	22.8 to 40.3	<0.001
Breast Reconstruction	48	31.5	20.2 to 42.7	<0.001
Hand Trauma Reconstruction	133	29	24.0 to 34.1	<0.001
Infection Control and Prevention	160	27.6	22.8 to 32.5	<0.001

Laparoscopic Handling of Small Bowel	8	26.3	1.8 to 50.7	0.039
Acute and Chronic Pain	40	25.3	15.8 to 34.8	<0.001
Oral Health in Comprehensive Cleft Care	32	25.2	14.1 to 36.3	<0.001
Chest Wall Reconstruction	9	25	−1.7 to 51.7	0.063
Ectopic Pregnancy	10	25	−8.9 to 58.9	0.13
Emergency Checklists in Anaesthesia Practice	89	23.7	17.2 to 30.2	<0.001
Trocar Placement	15	21.3	6.3 to 36.4	0.009
Penetrating Thoracoabdominal Trauma	31	20.7	10.0 to 31.3	<0.001
Common Intraoperative Problems	140	19.1	14.8 to 23.5	<0.001
Drug Errors in Anaesthesia	64	18.4	12.5 to 24.3	<0.001
Appendicitis	46	17.2	9.5 to 24.8	<0.001
Laparoscopic Cholecystectomy	19	15.3	2.1 to 28.5	0.026
